# Plasma-based COVID-19 treatments in low- and middle-income nations pose a high risk of an HIV epidemic

**DOI:** 10.1038/s41541-020-0209-2

**Published:** 2020-07-06

**Authors:** Leonardo M. R. Ferreira, Mohammed A. Mostajo-Radji

**Affiliations:** 1 Clubes de Ciencia Bolivia Foundation, Santa Cruz de la Sierra, Bolivia; 2 Department of Surgery, University of California San Francisco, San Francisco, CA 94143 USA; 3 Diabetes Center, University of California San Francisco, San Francisco, CA 94143 USA; 4 Ajax Biomedical Foundation, Newton, MA 02458 USA; 5 The Eli and Edythe Broad Center of Regeneration Medicine and Stem Cell Research, University of California San Francisco, San Francisco, CA 94143 USA; 6 Embassy of Science, Technology and Innovation, Ministry of Foreign Affairs of Bolivia, La Paz, Bolivia; 7 Permanent Mission of Bolivia to the United Nations, New York, NY 10017 USA

**Keywords:** Infectious diseases, Vaccines

## Abstract

Convalescent plasma therapy holds promise as a transient treatment for COVID-19. Yet, blood products are important sources of HIV infection in low- and middle-income nations. Great care must be taken to prevent plasma therapy from fueling HIV epidemics in the developing world.

The COVID-19 pandemic has inspired rapid research towards medications and vaccines to prevent the spread of the disease. Many drugs have been promoted as potential treatments for the disease, including hydroxychloroquine, azithromycin, Remdesivir, Avifavir, ivermectin, chlorine dioxide, among others^1^. Their effectiveness has been based on small studies and massively inflated by the media, only to later show limited to no benefit in larger studies and, sometimes, even cause severe side effects. The reality is that, to date, there is no proven drug to treat COVID-19. A potential vaccine will take at least 1 year to be developed and tested. Yet, a promising strategy to hold down the fort has arisen: convalescent plasma therapy. Trials to treat COVID-19 using this method are being initiated in many countries, including low- and middle-income nations in Africa, Southeast Asia, and Latin America.

Convalescent plasma therapy is based on the concept of passive immunity. Individuals who recover from SARS-CoV-2 infection have, in principle, developed neutralizing antibodies against the virus^2^. Collecting plasma, the liquid component of blood, from someone who has recently recovered from COVID-19 and infusing it into someone with an ongoing infection would confer the plasma recipient with antibodies to combat the virus^3^. Of note, this is an immediate transient treatment and does not replace the long-lasting immune memory generated by a vaccine. Indeed, high-affinity IgG antibodies have a half-life of up to three weeks in blood^4^. Nevertheless, the hope is that the infusion of convalescent plasma enriched in antibodies will substantially boost the recipient’s immediate immune response to clear the virus.

The infrastructure for collecting and administering plasma exists. The risks are known and rather low when the healthcare infrastructure is optimal. More than 16,000 patients at hundreds of US hospitals have received convalescent plasma therapy for COVID-19. A study in New York City found that convalescent plasma recipients had improved survival and less-supplemental oxygen requirements than control patients^3^. Yet, convalescent plasma therapy is not without its perils, especially in low- and middle-income nations with suboptimal healthcare infrastructures and less strict regulations. Blood transfusions can transmit blood-borne pathogens^5^ and lead to conditions such as transfusion-related acute lung injury and transfusion-associated circulatory overload^6^. In fact, blood transfusions have been shown to represent an important source of HIV infection in many low- and middle-income countries, being associated with positive HIV status^7^. It is unlikely that most low- and middle-income countries will be able to secure the blood supply by universal HIV testing^7^. Even when funding is provided, access to medical materials and supplies in the international market remains difficult for the developing world^8^.

For years, the United Nations Development Programme (UNDP) and the World Health Organization (WHO) have subsidized molecular testing in the developing world. One such program, the introduction of “all-in-one cartridge” systems for RNA sample isolation and PCR-based testing, has been used for HIV and tuberculosis diagnosis^9^. This technology has recently been approved for COVID-19 testing, although with significant delays in the delivery of reagents and supplies^10^. Importantly, this platform has a higher cost and lower throughput than other PCR-based approaches. It allows the processing of only up to four samples per run, severely limiting its utility for COVID-19 and HIV PCR-based testing in larger communities. Moreover, governmental laboratories with RNA testing capabilities are currently saturated in many low- and middle-income countries^11^, leaving administrators to decide between testing for COVID-19 or HIV.

Regulations worldwide have forbidden paid organ and tissue donations for decades. Yet, these measures remain far from effective, particularly, in the developing world^12^. The reality of plasma donations is similar. In Bolivia, for instance, although law 1716 forbids any kind of payment for tissue donation, advertisements requesting paid plasma donations are common, even in prime time TV and national newspapers^13^. Such strategy is likely to be successful, as media coverage has been shown to positively impact organ and tissue donation rates^14^. Some regions have circulating lists of infected individuals. Plasma donations are compensated with thousands of dollars, several times the local average monthly salary, and can be performed once a week. Patients who recover following convalescent plasma infusion are then encouraged to donate their plasma. In addition to resembling a pyramid-type scheme, such practice does not have a solid scientific rationale, as these patients are highly unlikely to have developed any neutralizing antibodies against SARS-CoV-2. In fact, even among individuals who recover from SARS-CoV-2 on their own, one-third have low or undetectable neutralizing antibody titers^2^. Such a plasma black market is reminiscent of events in the 1990s in China, where an HIV epidemic began with local pay-for-plasma schemes^15^.

Rapid and affordable antibody-based HIV and COVID-19 testing capacity must be escalated in the developing world. One possible strategy is to share plasmids with biomedical facilities in low- and middle-income countries, which could then locally produce the reagents for antibody-based testing, circumventing the high costs and waiting times associated with importing such tests from abroad^10^. Centers with a proven track record of testing for endemic contagious diseases in the developing world exist, some of them resulting from multinational collaborations with developed nations^16^. Furthermore, antibody-based tests generated in-house may be more accurate and cost-effective than commercial ones^17^, so sharing parts and reagents (e.g., plasmids, purified antibodies) may be more desirable than already assembled testing kits. Importantly, conventional antibody-based HIV testing may miss early infection^18^. Hence, methods to detect viral nucleic acids directly remain desirable. Recently developed CRISPR-based methods to detect specific viral RNA sequences amenable to lyophilization, long-term storage, and reconstitution on paper represent a promising approach to detect the presence of SARS-CoV-2 and HIV, both RNA viruses, in locations with little to no healthcare infrastructures^19,20^. More bilateral and multilateral scientific collaborations between high- and low- and middle-income countries should be encouraged to gradually foster productive scientific collaboration and build local biomedical infrastructure^21^.

In summary, convalescent plasma therapy holds promise as an emergency transient treatment for critical COVID-19 patients. Nevertheless, it is of utmost importance to emphasize that, unlike chemicals, such as hydroxychloroquine, which can only harm the individual taking them, plasma therapy can endanger entire communities. In scenarios of scarce blood-borne pathogen testing capacities, few enforced regulations, and widespread misinformation and disease stigma, unregulated convalescent plasma therapy may well become a recipe for a new HIV epidemic in the developing world.
